# Ten years of the Genomics of Common Diseases: “The end of the beginning”

**DOI:** 10.1186/s13059-016-1125-7

**Published:** 2016-12-09

**Authors:** Chris S. Haley

**Affiliations:** 1 MRC Human Genetics Unit, Institute of Genetics and Molecular Medicine, University of Edinburgh, Edinburgh, UK; 2 The Roslin Institute and Royal (Dick) School of Veterinary Sciences, University of Edinburgh, Edinburgh, UK

## Abstract

The 10th anniversary ‘Genomics of Common Diseases’ meeting was held in Baltimore, September 25-28, 2016. Professor Chris Haley reports from the meeting on progress and challenges in the field.

The first meeting in the Genomics of Common Diseases series was held just after the publication of the first successful genome-wide association analyses (GWAS), when optimism for the genomic dissection of common complex traits and diseases was high. Much progress has been made in the intervening period, so the 10th meeting provided a good opportunity to assess successes so far and the path ahead. Here, I do not attempt to summarise every presentation but rather to draw out some of the important themes as I saw them.

## The value of exome sequencing

The meeting was book-ended by presentations focusing on the value of whole exome sequencing (WES). The first Keynote speaker, Richard Lifton (The Rockefeller University, USA), illustrated the use of WES in the identification of alleles with major effect with a number of examples. He pointed out that almost all Mendelian conditions are underpinned by variation in the exome and the proteins thus affected are potential drug targets. He also commented that whilst whole genome sequencing (WGS) provides much more data, it also costs approximately four times more than WES and the functional consequences of variation outside the exome are much more difficult to predict.

At the end of the meeting, two speakers highlighted the value of WES in isolated populations for identification of deleterious major mutations where recent population bottlenecks and drift have led to enrichment of these types of variant. Adam Locke (Washington University School of Medicine, USA) reported analysis of data from 20,000 Finnish exomes which allowed the identification of many rare variants associated with cardiometabolic traits. These included premature stop, splice, frameshift and missense variants associated with creatinine, adiponectin, ApoA1 and lipoproteins. Rick Dewey (Regeneron Pharmaceuticals, Inc., USA) reported on his company’s efforts to exploit WES from both cosmopolitan and isolate populations. Echoing Professor Lifton’s arguments, their analysis of over 100,000 exomes found many such ‘natural experiments’, identifying both harmful and protective genetic associations with clinical traits, including variants in known drug targets.

## The UK Biobank

Whilst WES is proving invaluable for the identification of individual Mendelian variants with large effects, the major progress in detecting the heritable component of common complex disease over the last 10 years has been through the application of SNP genotyping arrays to GWAS using large population samples. Until recently the largest samples have been accessed through meta-analysis, but in the last year results from the first 150,000 genotyped individuals from the UK Biobank have become available. These latter data made a major contribution to the associations identified for many haematological traits presented by Tao Jiang (University of Cambridge, UK) and colleagues. The UK Biobank was also one of several studies contributing to the GWAS of reproductive ageing presented by John Perry (University of Cambridge, UK), which also found potentially causal associations of this trait with several cancers including breast and prostate cancer.

Perhaps the most novel example of the traits explored by GWAS that was presented at the meeting was from Cisca Wijmenga (University of Groningen, Netherlands). She demonstrated how careful analysis of the microbiome using metagenomic sequencing of stool samples could be combined with whole genome data to identify host factors associated with the abundance of various microbial species. Although as yet relatively modest in size compared to studies of other traits, major results were independently replicated and the study identified evidence of host-by-diet interactions and suggestive associations with loci involved in various traits and diseases.

## The space between common and rare variants

Clearly, GWAS is effective at locating common variants of small effect and WES is identifying *de novo* and very rare variants of much larger effect. However, there appears to be a gap in the spectrum of allele effect sizes between these two types of study that represent variants that are not yet effectively captured. Our own presentation at the meeting explored the variation for quantitative traits and diseases that is associated with SNPs and with pedigree. We concluded that about half the variation was associated with the genotyped SNPs and the remainder with genetic variation within families that is not yet captured by SNPs, even after accounting for family environmental effects. In addition there was evidence for interactions between genes and environment that might help to explain why heritabilities from twins sharing common environments seem consistently higher than those from other types of families. It may be possible to increase the proportion of variation captured by GWAS by incorporating information from ever rarer SNPs. However, my own conclusion is that individual rare variants with larger effects are likely to be confined within families and kinships, with their frequencies constrained to low frequencies by the impact they have on fitness. This will necessitate an increased focus on genetic variation associated with families and isolate populations, rather than the unrelated samples now commonly used.

## Combining reference and local population samples

Whatever the population sampling strategy, the step beyond SNP based studies of large population cohorts is to complement these with sequence data from the exome or the whole genome. As a first step this uses sequence data from reference populations combined with SNP data from the populations under study to impute ungenotyped common and rare variants across the whole population. Harm-Jan Westra (Harvard Medical School, USA) used this approach to impute additional variants into regions around loci previously detected in rheumatoid arthritis and type 1 diabetes case-control cohorts. This allowed them to conclude that there were multiple putative causal variants in several of the loci and single putative causal variants in just two of the loci.

Although this approach has proved successful in cosmopolitan populations, it has become clear that individual populations have their own rare variants that can only be effectively imputed by combining reference population with sequence data from local populations. Liping Hou (National Institute of Mental Health, USA) and colleagues illustrated this with whole genome sequence from 265 individuals from an Anabaptist reference sample. They found a substantial number of variants not found in the 1000 genomes reference panel, with 43,000 variants that were rare (<0.5%) in the panel but with frequencies above 5% in Anabaptists. Manuel Rivas (Broad Institute, USA) described a similar situation in Ashkenazi Jews, where exome sequencing revealed that many protein coding alleles are enriched in this population. This includes 128 potentially pathogenic alleles which may contribute to elevated levels of some diseases in Ashkenazi Jews, with for example elevated frequencies of 11 risk variants contributing an estimated 44% of the 4-fold increased risk of Crohn’s disease in this population.

## Whole genome sequencing

Despite these and other successes, it is clear that ultimately whole genome sequence from very large population cohorts will be generated, necessitating step up in computational resource to deal with the massive data that will be generated. Benjamin Neale (Massachusetts General Hospital Research Institute, USA) described the Hail software package that uses a distributed computing approach to tackle this problem. He described the application of this software to 14,000 individuals with exome or whole genome sequence data to identify a role for very rare disruptive variants in educational attainment.

Gonçalo Abecasis (University of Michigan, USA) also described software tools, in his case applied to the first ~50,000 whole genome sequences from the National Heart, Lung and Blood Institutes TOPMed programme. Results included the identification of 1.5 million putative missense SNPs and 39,000 potential loss of function SNPs in the first ~20,000 sequenced individuals. In a follow-up discussion, he suggested that the 30x coverage target is the result of multiple competing considerations, including challenges in using the Illumina sequencing platform efficiently at lower depths. In his view, an optimum strategy for research studies is likely to sequence samples at lower depths and then fill any gaps computationally.

One consequence of the various methods that increase focus on rare variants is that individual variants may be increasingly difficult to detect and replicate, especially those confined to specific populations or kinships. Bogdan Pasaniuc (University of California, Los Angeles, USA) described applications combining summary GWAS association statistics. One application, termed ‘local SNP heritability’ combines information from multiple SNPs in a region accounting for LD between them to produce a composite signal that may contribute to alleviating this problem, providing replication at the locus level rather than the SNP variant level.

## Future directions

The meeting gave several glimpses of where research might be heading in the future. Eventually inroads will have to be made into assigning function to the many thousands of variants of small effect identified for hundreds of traits and diseases by past and future GWAS, most of which are not protein-coding. Michael Levine (Princeton University, USA) pointed out that we should not assume that enhancers are optimised to maximise expression. From elegant work on sea squirts it was clear that there was a balance between optimising expression and its tissue specificity, such that enhancers that maximised expression lost some tissue specificity. This raises the possibility that both increasing and decreasing enhancer activity could have deleterious consequences.

Karen Mohlke (The University of North Carolina at Chapel Hill, USA) gave examples of exploring candidate SNPs for type 2 diabetes and cholesterol using high resolution mapping combined with data on chromatin status followed by *in vitro* functional assays to explore their effects on expression. In one example the effects of one variant on expression were consistent across models and with known biology. But in a second several variants in a haplotype seemed to show differential effects on expression, and results had to be reconciled with apparently conflicting data from the literature before the picture made sense.

In order to make substantial progress with this herculean task it will be necessary to supplement detailed analysis of individual associations with high throughput methods to identify and test associations and their biological causation. Olga Troyanskaya (Princeton University, USA) presented work using the deep learning based ‘DeepSEA’ framework. This algorithm attempts to learn the regulatory sequence code from large-scale chromatin-profiling data and predict the tissue specific chromatin effects of single nucleotide variants in order to prioritise variants for follow-up.

Arjun Krishnan (from the Troyanskaya group) leveraged information of the small proportion of loci known to be associated with ASD in a machine learning approach that harnessed a brain-specific gene-interaction network to identify key pathways. Using this information they predicted novel loci associated with ASD and could validate some of these in an independent sequencing study.

The group of Nancy Cox (Vanderbilt University School of Medicine, USA) group is one of several that have recently shown how GWAS associations with a trait can be used to impute that trait into populations where the trait is not recorded but whole genome SNP data is available. In the examples she presented, gene expression was imputed into the large BioVu cohort of individuals with electronic health record data, enabling inference on the potential association of locus expression with disease. For example SLC39A4 is a zinc transporter and known Mendelian loss of function variants in this locus lead to severe inflammatory rash associated with zinc deficiency that can be ameliorated by zinc supplementation. Predicted variation in expression of this locus was associated with similar symptoms in patients not carrying Mendelian loss of function variants, suggesting that zinc supplementation might benefit some of these patients. Consequently a “pragmatic trial” to test this hypothesis in the patient cohort is underway. This study thus provides a bridge showing how Mendelian variants, often driven by exonic variation as noted right at the start of the meeting, can help understand the impact of variants of smaller effect typically identified by GWAS which lie outside of the exome and have their impact via modulation of transcription.

The final presentation in the meeting by Kiran Musunuru (University of Pennsylvania, USA) gave a glimpse of how potential causative variants might be assessed in future using high-throughput phenotyping. He has used a CRISPR based approach to generate all catalogued coding variants in the cardiomyopathy-associated locus TNNT2. The aim is to generate mutant cell lines and use gene expression and phenotypic profiling to assess the pathogenicity of the variants. This approach could be used in future to rapidly assess novel patient-specific disease variants.

## Conclusions

Much has been achieved in terms of understanding the genetic basis of disease. But a great deal more remains to be done if we are to understand and exploit the underlying biological causes of all the associated variants and turn this knowledge into treatments. The first steps down this path have been taken and the meeting demonstrated much optimism for the future; but in reality we are only just reaching “the end of the beginning” with many more challenges lying ahead.

